# Hospital Meetings

**Published:** 1901-06-15

**Authors:** 


### HOSPITAL MEETINGS.

### LONDON HOSPITAL.

A quarterly Court of Governors of the London Hospital was held on the 5th instant, Mr. Sydney Holland, the chairman, presiding.

The annual report which was laid on the table stated that the year 1900 had been one of the very busiest in the history of the hospital. A great part of the central block had been out of use owing to the building going on, but not a single bed had been closed. The total number of in-patients treated was 12,746, and the total number of new cases seen in the receiving-room and in the out-patient department amounted to 161,762. The committee were able to report that the system introduced in June, 1898, by which all patients are seen at once, on presenting themselves in the receiving-room, without any governors' or subscribers' letter, had worked most satisfactorily. On being seen by the receiving-room officer, who is an experienced medical man specially appointed to fill this difficult post, the patient is either admitted for treatment as an in-patient (if this is considered necessary), or is referred to the proper department for further continuous treatment as an out-patient. At the same time the existence of the hospital as a charity giving help only to thoroughly deserving cases is most jealously guarded, and a strict inquiry made into the circumstances of all cases referred for continuous treatment to the out-patient department by one of the out-patient inspectors. The plan of charging 3d. to the out-patients for the medicine or bandages supplied to them had also been found to work satisfactorily, and the amount received by the hospital during the last year from this source was £1,317, but of the total expenditure of the dispensary last year, over £6,000 was due to the treatment of out-patients. No charge is made for those who are found to be too poor to pay, or for children of school age and under.

The work of the out-patient department had been considerably extended during the past year by the institution of the light cure for lupus. Her Majesty Queen Alexandra, recognising the benefits of this method of cure carried on in her own native land by Dr. Finsen, was anxious that it should be available for the poor of London, and generously presented that hospital with a complete set of apparatus for the treatment of lupus cases. A part of the temporary ward, which had been erected in the garden to accommodate patients during the re-arrangement of certain portions of the old building, was set apart, and the light put up. So great was the influx of patients that it was found necessary by the committee almost immediately to instal a second light, and they now had a third lamp in contemplation which would be erected as soon as the necessary arrangements had been made. The treatment was slow, but certain, and practically painless. Patients had to attend every day for an average of three to four months. Last year a capital sum of £999 was expended, and the cost of the maintenance of the light from the date of its installation to the end of the year (7 months) was £641. Fortunately, the whole of this did not devolve on the charity, for though three hours in the morning were devoted to the treatment of poor patients who could not pay at all, they were able to extend the benefits of the treatment for two hours every afternoon to certain others who could afford to pay the charge made by the hospital, and this brought in £347 from the time they began the treatment of paying patients to the end of the year.

The average stay of patients during the year was 19·8 days, a slight increase; the average number of patients resident in the hospital each day was 657. The largest number of patients in the wards on any one day was 728. The ordinary expenditure for the year was £79,905, as compared with £72,629 in 1899, and the extraordinary expenditure—chiefly on the rearrangement of the hospital under the improvement scheme—£35,359; together, £115,264. It would be remembered that the Prince of Wales's Fund gave £5,000 per annum on condition that £100,000 was spent on certain alterations. Under that scheme there had already been spent: In 1898, £12,165; in 1899, £15,330; and in 1900, £27,187. The ordinary income was £62,530, and the extraordinary income, £40,084. This included £10,951 received from the sale of land in Charlotte Street to the Rowton Houses Company, and £15,335 from legacies. Legacies in the previous year yielded £27,158. In 1899 there was a falling off in the receipts of the people's subscription fund, but this year the income had risen from £1,571 to £1,658. To enable small contributors to help, the "Forget-me-not" Bond had been instituted, and it was hoped to get out at least 50,000 books, each representing a steady income of a penny per week. The committee felt that every possible opportunity should be given to working men to do all they could to supplement the gifts of those better off. Two gentlemen, who wished to remain anonymous, were contributing £25,000 and £13,000 to the cost of building the out-patient department and the new theatres ; and during the year £6,000 and £2,500 respectively had been received to meet outlay already incurred. Another donor, who also desired to remain unknown, had supplemented the £22,000 he gave for the isolation block by a further £10,000 towards the completion and furnishing of it. Mr. J. V. Hora had transferred a further £4,000, making a total of £6,000 towards the endowment of the " Marie Celeste " ward.

The CHAIRMAN moved the adoption of the quarterly report to date. He said :—By the annual reports which have been completed for issue since the last meeting of the Governors, it will be seen that there has been a slight falling off in the actual number of patients admitted during the year—12,154 as against 12,603 in the previous year. The expenditure has, however, been somewhat higher, with the result that the average cost per bed works out at a few shillings over the £100. It is felt by the committee that this result is extremely satisfactory considering the difficulties under which the work in the hospital has been carried on during the twelve months, and that all connected with this work are to be congratulated on the manner in which it has been dealt with in spite of the alterations and improvements which are going on. The new post-mortem room is now completed and is in full working order. The whole is a most handsome structure and is found to answer the needs of the hospital admirably. We have one of the best, if not the very best building for this special work in the kingdom ; and in addition there is a special room set apart where the friends of those who have died in the hospital may pay their last tribute of respect. The first part of the improved main building is now in occupation, as the steward has moved from his temporary rooms to his new offices ; and the new part of the receiving room is ready for use whilst the old part is undergoing slight alterations, and very shortly we shall have the whole in use. The new stores are on the eve of completion, and as soon as possible will be occupied. The new isolation block which is being built on the plot of land adjoining St. Philip's Church, is practically completed, and the lighting and fitting up are now being attended to. We have also completed the arrangements for the purchase of the John Baker's Almshouses, and as soon as the plans are completed it is proposed that this site should be utilised for the much needed laundry, when the present incommodious room will be cleared out from the basement of the east wing of the hospital building. In special reference to the work of the past quarter attention must be called to the fact that the number of patients has been extremely heavy, and on April 11th we had the largest number (737) of in-patients that have ever been in the hospital on any one day. Previous to that date the largest number was 733, in 1876. On census day the actual number of people returned in the Hospital was 982—424 males and 558 females—but it must be remembered that a large part of the staff, including the steward and some hundred or so nurses, are now living in houses which we have been obliged to take in Newark Street, Philpot Street, and Turner Street, so that it is safe to say that the actual number of people daily resident under the jurisdiction of the hospital is well over 1,000. As Dr. Hewitt is about to retire it has been arranged that he should be appointed consulting anæsthetist to the Hospital, and Emeritus Lecturer on the administration of anæsthetics. It has been decided to appoint three anæsthetists. The Governors will have noted with pleasure that Sir Frederick Treves has been appointed a K.C.V.O. The surgical registrarship having fallen vacant at the end of March, Mr. Newland was appointed to fill the post for the ensuing year, and on the expiration of his two years' term of office Dr. Andrews has been re-appointed for three months as obstetrical registrar. Mr. Dean having returned to his hospital work thoroughly restored to health, an extra day has been arranged for in the out-patient department and Mr. Barnard will see surgical out-patients on Friday afternoons. It is not possible to arrange for more surgeons to attend at the present time as all the rooms are now occupied fully every day, practically all day, and the time when the new out-patient department, which is already showing vigorous signs of growth, is completed and ready for use, is looked forward to by all who have to deal with the heavy number of out-patients who come here. Mr. Openshaw has also arranged, with the full concurrence of the committee, to see some of his special orthopædic cases on Saturday mornings. It is necessary to mention the great success that has attended the treatment of lupus by light. So numerous have been the applications that though the third light was opened for use on May 22nd, there was very little hope of our being able to deal with all the sufferers. We have had a most generous gift of £10,000 from Mr. Alfred Harmsworth for the endowment of this third light, and the burden of anxiety with which the committee undertook this costly, though most beneficial treatment, has thus been considerably lightened. It is hoped that the noble example of Mr. Harmsworth may secure further help from others, as we have still two lamps requiring endowment, the cost of running which is £400 a year each. We are glad to report that lamps will shortly be opened in Manchester and Liverpool.

It is with the deepest regret that the committee have to report to the Governors the death of Sister Lloyd at Pretoria. She went out as a sister specially chosen by the Queen for service in South Africa as one of the Princess of Wales's Nurses. This is the second death which has occurred among the loyal band of workers who went out to cope with the terrible work of attending to our soldiers in South Africa. Miss Lloyd was one of the best sisters we had, and her loss is very greatly mourned at the hospital. Dr. Sansom has been obliged, after many years, to give up the medical care of the sick nurses, and Dr. F. J. Smith has kindly undertaken this duty. Mr. Hutchinson, who has been lecturer on surgical nursing to the nursing staff for five years, has been obliged, through the increased demands of his other work, to give up these lectures. Most cordial thanks were sent to Mr. Hutchinson by the house committee for the able manner in which he has conducted these lectures.

After notifying the receipt during the quarter of legacies amounting to £4,326, and mentioning that owing to the heavy strain caused by the building operations the committee had to borrow £20,000 to meet the accounts due last quarter, the CHAIRMAN referred to the announcement of a Victoria Cross having been conferred on Mr. Neville Howse, who before going to Australia had been a London Hospital student and one who did them the greatest credit, having held there all the appointments he could hold. Mr. Holland then dealt with what had been described as a " Hospital Scandal." He said he supposed they would be delighted if they could find out something scandalous about the Archbishop of Canterbury, and the greater and more beneficent an institution was, the more ready some people seemed to believe anything bad about it. It was not true that a woman had remained from nine in the morning till ten at night without attendance: such a thing was impossible. The woman was taken into the receiving room, where her wounds were properly dressed by the senior resident medical officer, and she was then put to bed and left peacefully in charge of the sisters and nurses. Later the surgeon saw her, administered an anæsthetic—anæsthetics should, of course, be administered on an empty stomach—and stitched up the wounds. He saw her again at 2.30, at 9, and at 10. As to the complaint that they had not called in the police, it was no part of their duty to go round to the poor people who were brought in to them, making inquiries as to how they came by their injuries. There were other people who did know the circumstances, but as a fact when the change for the worse in this woman's condition took place, they did telephone to the police about it. He concluded by moving the adoption of the report.

This was seconded by Mr. Spencer Charrington, M.P. and carried *nem. con.* Formal business concluded the proceedings.

